# Photoelectrocaloric effect in ferroelectric oxide

**DOI:** 10.1038/s41598-022-10331-8

**Published:** 2022-04-16

**Authors:** Subhajit Pal, Manu Mohan, K. Shanmuga Priya, P. Murugavel

**Affiliations:** Functional Oxides Research Group (FORG), Department of Physics, Indian Institute of Technology Madras, Chennai, 600036 India

**Keywords:** Materials science, Condensed-matter physics

## Abstract

The enhanced electrocaloric (EC) effect in solid-state-based lead-free ferroelectric Ba_0.875_(Bi_0.5_Li_0.5_)_0.125_TiO_3_ system is investigated under light as an external stimulus. The sample exhibits an analogous value of maximum change in entropy at Curie temperature, extracted from the two different measurements process. Notably, the sample depicts maximum value of adiabatic change in temperature (Δ*T*) as 1.27 K and isothermal entropy change (Δ*S*) as 2.05 J/K kg along with the EC coefficient value of 0.426 K mm/kV, under dark conditions. In addition, the sample exhibits > 0.5 K adiabatic temperature change over a broad temperature range (~ 35 K). Remarkably, the EC parameters display ~ 27% enhancement upon 405 nm light illumination. The demonstrated photoelectrocaloric effect is found to be in accordance with theoretical formalism. The present work elucidates the light as an additional degree of freedom to widen the potential of solid-state-based technologies for advanced environment-friendly cooling devices.

## Introduction

Search for materials having potential usage in the forefront of alternative energy and related applications are vital for future technology. The cooling devices, which consume a giant part of global energy, are currently based on inefficient and non-eco-friendly vapor compression technology^1,2^. Efforts are made to develop environment-friendly solid-state cooling technologies as an alternative to the existing refrigerant^3–5^. In this context, pyroelectric materials are getting attention due to their large electrocaloric (EC) effect^6,7^. In particular, ferroelectric compounds showing a large pyroelectric response near phase-transition temperature (*T*_C_) could be envisaged as a potential candidate for modern EC devices^3–8^. There are proposals reported in the literature to improve the EC response in ferroelectrics by geometrical optimization^9^, modifying the energy landscape among the coexisting phases^10^, integrating the positive and negative caloric responses^11^, and incorporating additional degrees of freedom^12,13^.

In addition, applying external degrees of freedom such as electric field, mechanical stress, strain gradient, and pressure reveal significant enhancement in the EC effect of ferroelectric systems^12–17^. For example, the electric field-induced enhanced EC effect with tunable characteristics is reported in PbMg_1/3_Nb_2/3_O_3_–PbTiO_3_ near room temperature^16^. Additionally, the phenomenological model suggests the possibility of tuning the *T*_C_ at which the EC effect is maximum under hydrostatic pressure^12^. In fact, this idea has been experimentally verified on metal-free ferroelectric [MDABCO](NH_4_)I_3_ sample, where the maximum observed EC response is reported to shift from 450 to 293 K^17^. Also, lattice strain-induced EC enhancement is theoretically predicted on SrRuO_3_/BaTiO_3_/SrRuO_3_ thin film^14^. Recently, there are reports to couple the EC effect with flexoelectric efffect originated from the strain-gradient engineered in thin film samples^18^. Note that the extent of enhancement and tunable characteristics of the EC effect can vary with types of external stimuli.

In this context, the reported light-induced phenomena on the ferroelectric systems such as photovoltaic effect, photostriction, and photoferroelectric effect highlight the strong correlation between the polarization dynamics with light^19–22^. In particular, photoferroelectric effect is attributed to the photo-induced changes in polarization dynamics caused by altering the surface screening effect^21,22^. Since the EC response also depends on the polarization dynamics of the material, it is envisaged to enhance its performance characteristics using light as an external stimuli. To explore such light-induced EC response, lead-free ferroelectric Ba_0.875_(Bi_0.5_Li_0.5_)_0.125_TiO_3_ (BBLT) compound exhibiting a giant photovoltaic response is considered as a model system^23^. The studies carried out under dark and light conditions revealed a remarkable ~ 27% enhancement in the EC effect. The photoelectrocaloric effect presented in this work demonstrates the application of light as an additional degree of freedom to tune the EC response suitable for advanced solid-state cooling devices.

## Materials and experimental techniques

The BBLT compound is synthesized by a conventional solid-state reaction method using stoichiometric mixtures of analytical reagent-grade BaCO_3_ (99.9%), Bi_2_O_3_ (99.9%), Li_2_CO_3_ (99.9%) and TiO_2_ (99.9%) powders by following the synthesis conditions reported earlier^23^. The pellet made from the calcined powder is sintered at 1000 °C for 2 h and the density of the pellet is found to be 96% of the theoretical value.

The X-ray diffraction (XRD) experiment is carried out by the Rigaku X-ray diffractometer for the structural information. The temperature-dependent dielectric measurements at various frequencies from 100 Hz to 1 MHz are obtained using Nova Control (Alpha-A) high-performance frequency analyzer. For optical bandgap, a diffused reflectance spectroscopy experiment is performed using Ultraviolet–Visible-Near Infrared (Jasco V-650) spectro-photometer. The pyroelectric measurements are performed on 8 mm diameter and 300 μm thick pellet in a closed-cycle cryostat (Advance Research System) using Keithley electrometer (6517B) as a current measuring unit. The polarization versus electric field measurement is carried out using Radiant Technology loop tracer at 300 K. The polarization measurement under illumination is performed on 12 mm diameter and 0.2 mm thick sintered pellet. The heat capacity is carried out by a Quantum Design (DynaCool-D212) physical properties measurement system. Light-induced pyroelectric measurements are carried out by employing a 405 nm diode laser (MDL-III-405) as a light source having 1.5 mm beam diameter. For light-induced measurements, 1.0 mm diameter Ag dots are used as top electrodes with Ag coating as a bottom electrode. The power density of the incident light is measured using a Coherent PM-10 power meter. To verify the change in temperature upon light illumination, the IR images are taken at room temperature under dark and light conditions using MIKRON (HT7600M) camera having 0.1 °C minimum sensing capacity in auto focusing mode. The images clarify that the temperature of the sample remains unaffected by the light within the measurement limit.

## Results and discussions

**Figure 1:**
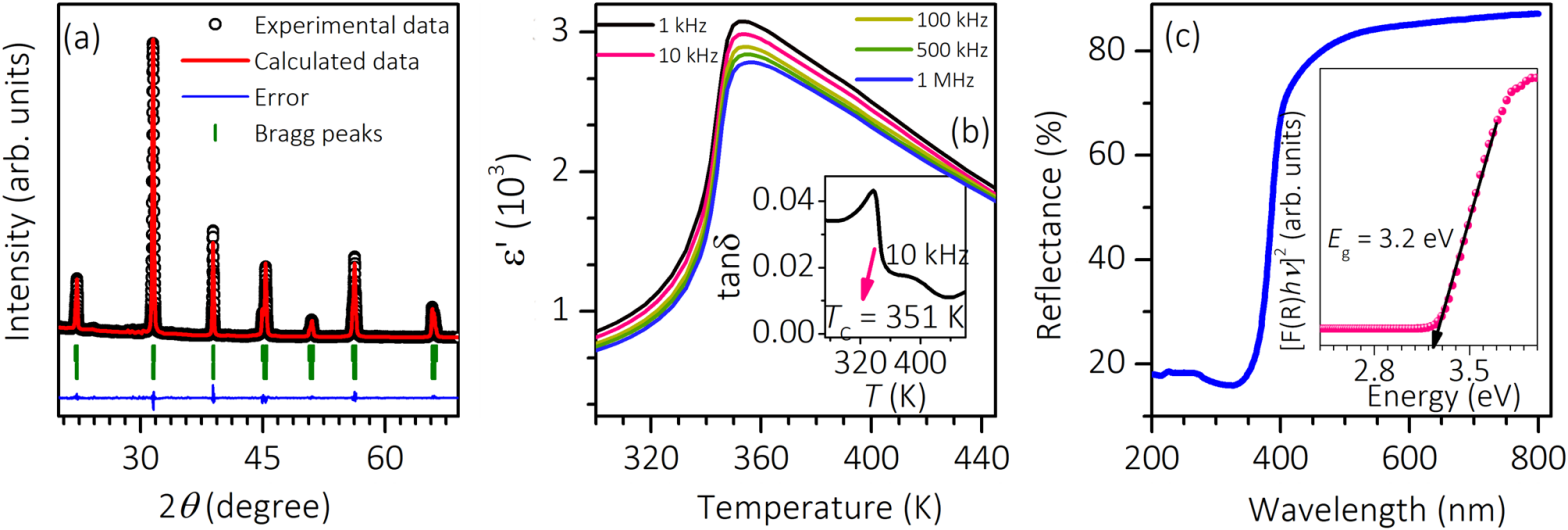
Stutural, dielectric and optical properties of BBLT. (**a**) Reitveld refined XRD pattern recorded at 300 K and (**b**) temperature-dependent *ε*′ plotted at different frequencies for the BBLT sample. The inset shows the dielectric loss factor at 10 kHz. (**c**) The reflectance spectrum of the BBLT sample. The inset shows the corresponding Kubelka–Munk plot.

The XRD pattern shown in Fig. 1a indicates the formation of the BBLT compound, which is free from impurity phases. The obtained pattern is subjected to Rietveld refinement. The obtained goodness of fit (*χ*^2^ = 2.07), and the weighted profile factor (*R*_wp_% = 8.13) suggest the satisfactory fitting. The refinement shows the coexistence of tetragonal (*P*4*mm*) and orthorhombic (*Amm*2) phases with 84.79% and 15.21% phase fractions, respectively. The temperature-dependent real part of permittivity (*ε′*) measured at 1 kHz to 1 MHz frequency range is plotted in Fig. 1b. The plot unveils the ferroelectric to paraelectric transition (*T*_C_) at 351 K, and it is further verified from the temperature-dependent tangent loss (tan$\delta$) measured at 10 kHz, shown as an inset in Fig. 1b. The value of tan$\delta$ over the temperature range indicates that leakage contribution is insignificant in the sample. A noticeable smeared nature of *ε*′ near *T*_C_ indicates that the transition could be associated to diffuse phase transition^24^. However, frequency-independent *T*_C_ seen in Fig. 1b shows a non-relaxor characteristic of the BBLT sample. To obtain the optical bandgap, the diffused reflectance spectrum recorded on the sample is shown in Fig. 1c. The corresponding [F(R)*hν*]^2^ versus *hν* plot is shown as an inset in Fig. 1c, where $\mathrm{F}\left(\mathrm{R}\right)= \frac{{\left(1-\mathrm{R}\right)}^{2}}{2\mathrm{R}}$ is the Kubelka–Munk function, R is the reflectance, *h* is the Planck’s constant, and *ν* is the frequency^25^. The bandgap of the sample extracted from the plot is 3.2 eV.

**Figure 2:**
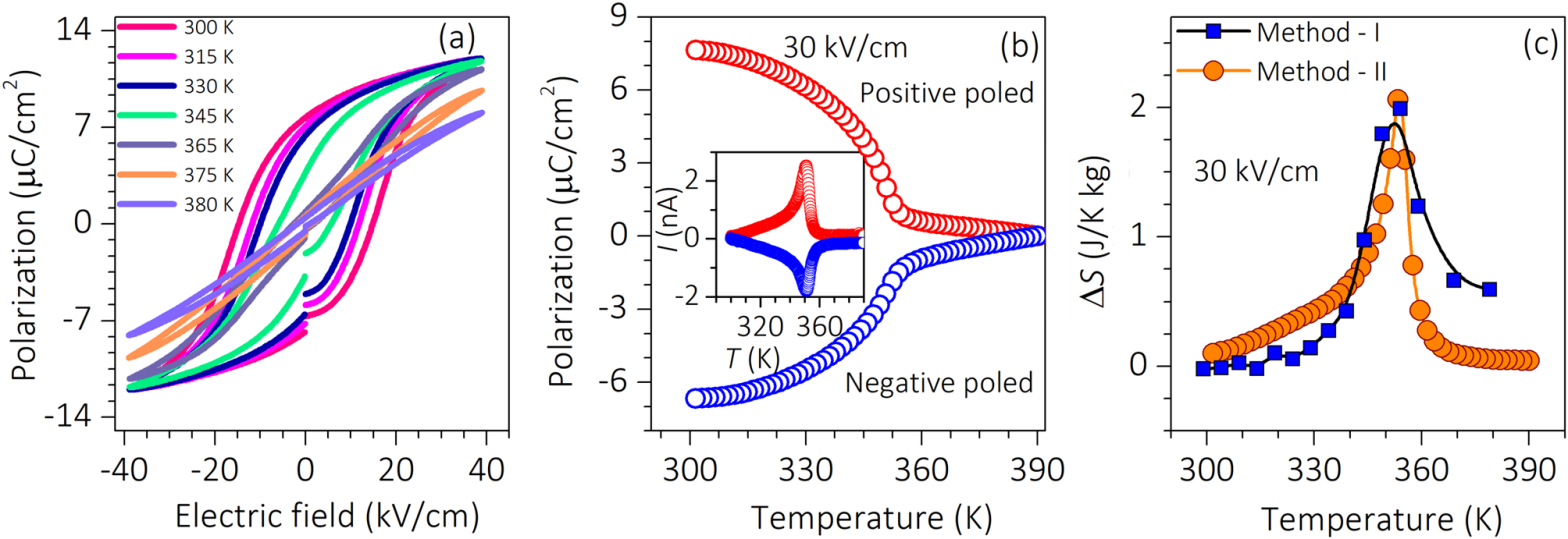
Temperature-dependent *P* and Δ*S* measurements of BBLT sample. (**a**) *P–E* hysteresis loop at different temperature. (**b**) Temperature-dependent *P* extracted from the pyroelectric measurements at positive and negative poled states. The inset shows the respective pyroelectric current response. (**c**) Δ*S* extracted from pyroelectric and polarization methods.

To investigate the EC response in the BBLT sample, the indirect method is adopted. To obtain the EC parameters, the temperature-dependent polarization data are extracted from two different approaches; Method (I): polarization (*P*) versus electric field (*E*) and Method II: pyroelectric current measurements^4,7,10,26,27^. For this purpose, temperature-dependent *P*–*E* measurement from 300 to 380 K recorded on the sample at 4 Hz is shown in Fig. 2a. The *P–E* hysteresis at 300 K reveals the typical hysteresis loop depicting the switchable polarization characteristics of the ferroelectric system. Temperature-dependent pyroelectric current measurement is performed to extract the corresponding *P* values. Prior to the pyroelectric current measurements, the sample is heated up to 390 K without any field and then cooled down to 300 K at 5 K/min under 30 kV/cm positive poling field. Then the pyroelectric current is measured during the heating cycle by maintaining the heating rate at 5 K/min in the absence of poling field. The experiment is repeated but this time under the negative poling field of same magnitude. The polarization values are extracted from the observed pyroelectric current using $P= \frac{1}{A\beta }\int i dT$, where *A*, *i*, *β*, and *dT* are the surface area, pyroelectric current, heating rate, and change in temperature of the sample, respectively^28^. The temperature-dependent *P* values extracted under positive and negative poled states are plotted in Fig. 2b. The respective pyroelectric currents are shown in the inset of Fig. 2b. The symmetric nature of the *P* curves under positive and negative poled states indicates the ferroelectric characteristics of the sample. The *P* value of the samples extracted from the pyroelectric measurement is ~ 7.5 µC/cm^2^ at 300 K, which is nearly matching with the value (8.3 µC/cm^2^) obtained from the *P-E* hysteresis loop. The *P* shows a decreasing trend with temperature followed by a drop in value near the vicinity of *T*_C_. Also, the *P* response shows nearly saturating features over a narrow range of temperatures. These observed features could be attributed to the diffuse-phase transition characteristics and structural inhomogeneity in the sample, reported in several ferroelectric systems^29–31^.

To extract the EC characteristic parameter, the isothermal entropy change (Δ*S*) and adiabatic temperature change (Δ*T*), the respective thermodynamic Maxwell’s equations $(\partial P/\partial T)_{E} = \left( {\partial S/\partial E} \right)_{T}$, and $\left( {\partial T/\partial E} \right)_{T} = ~ - \frac{T}{{C_{P} \rho }}~\left( {\partial P/\partial T} \right)_{E}$ are used^4^. The EC characteristic parameters can be expressed as $\Delta S=-\frac{1}{\mathrm{A}\beta \rho }i(T)\Delta E$, and $\Delta T=- \frac{T}{A\beta {C}_{P}\rho }i(T)\Delta E$^26,27^. Here, $i\left(T\right)=A \frac{\partial P}{\partial t}=A \frac{\partial P}{\partial T} \frac{\partial T}{\partial t}=A\gamma \beta ,$ where $\frac{dP}{dT}= \gamma$ is a pyroelectric coefficient, *ρ* and *C*_*P*_ are mass density and heat capacity, respectively^26,27^. The $\Delta E={E}_{1}-{E}_{2}$ is the difference in initial (*E*_1_) and final (*E*_2_) poling fields. For comparison, the pyroelectric coefficient $\gamma$ is calculated from both temperature-dependent *P*–*E* hysteresis (Method-I) and pyroelectric current (Method-II) measurements. The corresponding Δ*S* is extracted from the Maxwell’s equation by considering the poling fields lower limit *E*_1_ = 0. The obtained Δ*S* from Method-I and Method-II as a function of temperature are plotted in Fig. 2c. The Δ*S* obtained from both the methods shows an increasing trend with temperature below *T*_C_, and displays maxima at *T*_C_. The calculated Δ*S* at *T*_C_ (351 K) by the Method-I and Method-II are 1.99 and 2.05 J/K g at 30 kV/cm, respectively.

**Figure 3:**
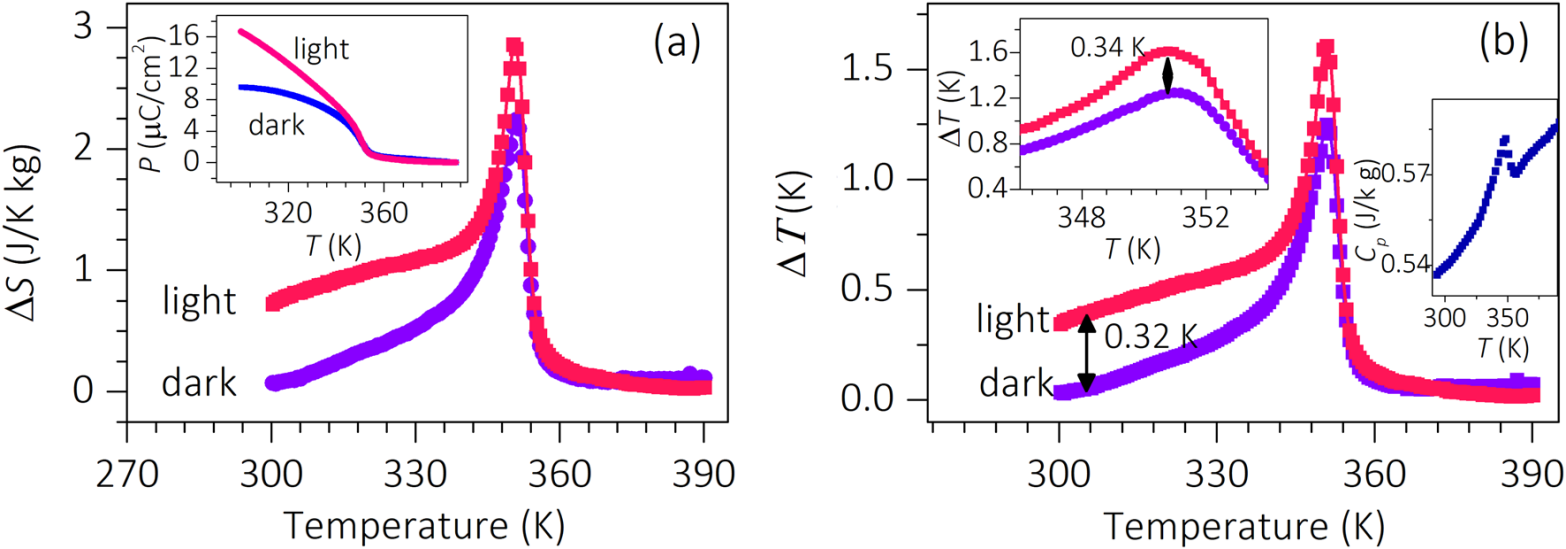
Photoelectrocaloric effect. Temperature-dependent (**a**) Δ*S* and (**b**) Δ*T* of BBLT sample measured under dark and light illumination conditions at + 30 kV/cm field. The insets show the respective *P* versus *T* curve, an enlarged version of Δ*T* versus *T* plot near *T*_C_, and temperature-dependent heat capacity of the sample.

Looking at the perspective of the reported light-induced effect on ferroelectric characteristics of the non-centrosymmetric system, it would be interesting to study the EC phenomenon in the BBLT sample under the influence of light^21,22,32–36^. To investigate it, pyroelectric measurement is repeated under the illumination of 405 nm with 11.9 mW/mm^2^ intensity of light at 30 kV/cm poling field. The corresponding temperature-dependent polarization graphs under dark and light illumination conditions are plotted in the inset of Fig. 3a. As the Δ*S* obtained from the two methods is comparable in value, Method-II is chosen owing to its ease in performing the experiment under light. The graphs exhibit enhanced polarization characteristics of the sample upon light illumination. This is in accordance with the variation shown by the *P-E* hysteresis loops measured under dark and light illumination conditions displayed in Fig. S1a (Supplementary information). The obtained *P*_r_ value plotted as a function of light intensity in Fig. S1b (Supplementary information) depicts the linear variation of *P*_r_ with light intensity. The unsaturated feature in polarization response derived from the pyroelectric current measurement below transition temperature could be due to the leakage current contribution upon light illumination. However, the intrinsic origin of the phenomenon can be elucidated from the light-enhanced dielectric characteristics with negligible tanδ variations observed on BBLT sample^22^. In fact, a theoretical model is also proposed by V. M. Fridkin on the origin of photoferroelectrics, where the effect is correlated to the change in surface screening conditions associated to the trapped and surface charges^21^. Few mechanisms are also proposed to explain the photoferroelectric effect in the material. For instance, the increase in *P*_r_ in (K_0.49_Na_0.49_Ba_0.02_)(Nb_0.99_Ni_0.01_)O_2.995_ sample is observed by Bai et al. which is claimed to be originated from the ferroelectric domains contribution^34^. Importantly, the contributions of ferroelectric domain and lattice deformation on the photoferroelectrics properties are also demonstrated by Pal et al. in the BBLT system^22^. Consequently, enhanced dielectric and ferroelectric characteristics are reported on several ferroelectric systems under light illumination, including BBLT, (K_0.49_Na_0.49_Ba_0.02_)(Nb_0.99_Ni_0.01_)O_2.995_,and Pb[(Mg_1/3_Nb_2/3_)_0.68_Ti_0.32_]O_3_ systems^21,22,34–36^.

To investigate the photoelectrocaloric response, Δ*S* and Δ*T* derived using Maxwell’s equations under dark and illumination conditions are displayed in Fig. 3a,b. Prior to that the specific heat *C*_*P*_ is measured, and the temperature variation of *C*_*P*_ is displayed in the inset of Fig. 3b. Interestingly, the EC response plotted in Fig. 3 illustrates significant changes in both Δ*S* and Δ*T* values throughout the temperature of measurements upon light illumination conditions. The maximum changes are seen near *T*_C_, where the Δ*S* and Δ*T* are enhanced from 2.24 to 2.87 J/K kg and 1.27 to 1.61 K, respectively. It is noteworthy to mention that the sample exhibits ~ 27% enhancement in EC response under light illumination condition. In addition, the *T*_C_ of the BBLT sample is decreased to 349 K under light illumination. This could be correlated to the photo-generated non-equilibrium carriers, which facilitate the transition to happen at a lower temperature^21,22^. Importantly, the observed Δ*T* is maintained above 0.5 K over a broad range of temperature (~ 35 K) under light illumination. This could be attributed to the diffused phase transition characteristic associated with the ferroelectric to paraelectric transition in BBLT sample. It is noteworthy to mention that the observed EC effect in the sample is superior compared to the reported results on other BaTiO_3_-based ferroelectric systems measured by indirect method, as evidenced from the comparison table given in Table 1. Table 1 also emphasizes the increase in EC coefficient (Δ*T*/Δ*E*) 0.42–0.54 K mm/kV at 30 kV/cm under dark and light illumination conditions. The detailed comparison of the EC effect observed in several ferroelectric systems is tabulated in Table 1.

**Table 1:** The comparison of EC characteristics of BaTiO_3_-based ferroelectric systems obtained from the indirect measurements.

Systems	*T*_C_ (K)	*E*_max_ (kV/cm)	Δ*T* (K)	Δ*T*/Δ*E *(K mm/kV)	*T* span (K)	*P*_r_ (μC/cm^2^)
BaTiO_3_^37^	403	55	0.78	0.142	40 (Δ*T* ≈ 0.6 K)	~ 12
0.98BaTiO_3_-0.02(BiMg_1/2_Ti_1/2_)O_3_^37^	416	55	1.21	0.220	50 (Δ*T* ≈ 0.9 K)	~ 9
Ba_0.85_Ca_0.15_Ti_0.90_Hf_0.10_O_3_^38^	396	35	0.74	0.211	75 (Δ*T* ≈ 0.6 K)	~ 7
Ba_0.94_Sm_0.04_TiO_3_^39^	347	30	0.92	0.306	7 (Δ*T* ≈ 0.8 K)	~ 9
BaTi_0.885_Sn_0.105_O_3_^40^	303	20	0.61	0.305	27 (Δ*T* ≈ 0.5 K)	~ 7
(Ba_0.835_Ca_0.165_)(Zr0_.09_Ti_0.91_)O_3_^41^	404	12	0.46	0.38	15 (Δ*T* ≈ 0.4 K)	~ 11
BaTi_0.89_Hf_0.11_O_3_^42^	343	10	0.35	0.35	31 (Δ*T* ≈ 0.2 K)	~ 6
0.9Ba(Ti_0.89_Sn_0.11_)O_3_-0.1(Ba_0.7_Ca_0.3_)TiO_3_^10^	323	40	0.91	0.35	30 (Δ*T* ≈ 0.6 K)	~ 8
(Ba_0.85_Sr_0.15_)(Sn_0.05_Ti_0.95_)O_3_^43^	309	20	1.44	0.52	NA*	~ 4
Ba(Ti_0.8_Zr_0.2_)O_3_–(Ba_0.7_Ca_0.3_)TiO_3_^44^	363	30	1.32	0.43	50 (Δ*T* ≈ 0.6 K)	~ 9
0.98BaTiO_3_-0.02NaNbO_3_^45^	350	200	3.6	0.18	35 (Δ*T* ≈ 3.2 K)	~ 4
BBLT ^Present work (Dark)^	351	30	1.27	0.426	13 (Δ*T* ≈ 0.5 K)	~ 15.8
BBLT ^Present work (Light)^	349	30	1.61	0.536	35 (Δ*T* ≈ 0.5 K)	~ 8.3

Here mentioned *P*_r_ values are measured at room temperature.

*NA not available.

To rule out the optical heating effect, if any, IR images recorded on the sample surface before and after 20 min of light illumination are provided in Fig. S2 (Supplementary information). Figure S2 depicts that the sample did not show any appreciable change in temperature before and after light illumination. This eliminates the possibility of optical heating effect on the EC response of the BBLT sample. To ascertain it further, the photographic images of the temperature controller (having sensitivity of 0.001 K) displaying the temperature of the sample under dark and light illumination conditions at different timing are shown in Fig. S3 (Supplementary Information). In this case, the thermocouple is connected to the bottom of the sample and the light is shined from the top the samples. Top and bottom sample surfaces are linked via thermally conductive silver paint. Note that 30 min of light illumination caused only 0.092 K change in temperature on the surface, validating the negligible optical heating effect. In addition, if it is believed that the variation in Δ*T* under light illumination condition is caused by the optical heating effect, then the variation in Δ*T* is expected to be noticeable even above *T*_C_. But, negligible variations in Δ*T* observed above *T*_C_ under light illumination conditions rule out the heating effect on the EC response throughout the measured temperature range, as displayed in Fig. 3b. Hence, the photoelectrocaloric effect observed on the BBLT sample is indeed an intrinsic property of the ferroelectric system.

The correlation between Δ*S* and *P* under the influence of light can be understood from the free energy perspective^21,22^. After solving the free energy equation, the spontaneous polarization of a ferroelectric system under light can be expressed as, ${P}_{0N}^{2}$ = ${P}_{0}^{2}\left[1 + \frac{bN}{\beta } - \frac{cN}{\gamma }\right]$ where ${P}_{0}$, *b*, and *c* are constants representing ferroelectric polarization, fourth, and sixth-order partial derivative of total energy near *T*_C_, respectively^21^. *N* is the energy level, *β,* and *γ* are coefficients in the free energy expression^21^. In the first-order phase transition, the Δ*S* can be expressed as, $\Delta S=1/2\alpha {P}_{0}^{2}$, here $\alpha$ is a phenomenological coefficient^46^. However, under light illumination, the Δ*S* is expected to show the enhancement upon replacing ${P}_{0}$ by ${P}_{0N}$. These indeed validate the observed photoelectrocaloric effect in the BBLT sample. Similarly, the light-induced change in Curie temperature $\Delta$*T*_C_ ﻿is expressed as $\Delta {T}_{C}={T}_{CN}-{T}_{C}=-\frac{C}{2\pi }aN$, where *C* is the Curie–Weiss constant, *a* is constant representing second order partial derivative of total energy with respect to polarization near the phase transition temperature, and *T*_C*N*_ is the Curie points under the light^21^. The minimum free energy condition gives *a* > 0, and hence the light-induced charge carriers are expected to lower the *T*_C_.

## Conclusion and outlook

In summary, the dielectric and pyroelectric measurements on lead-free BBLT sample revealed diffused phase transition near *T*_C_ and switchable polarization characteristics, respectively. The EC studies on the BBLT sample carried out at different poling fields displayed the maximum of Δ*S* = 2.05 J/K kg and Δ*T* = 1.27 K at 30 kV/cm field. The sample revealed remarkable ~ 27% enhancement in EC response under 405 nm light illumination with Δ*S* = 2.87 J/K kg and Δ*T* = 1.61 K at 30 kV/cm. Though the EC response is observed throughout the measurement temperature range, it is noteworthy to mention that the Δ*T* > 0.5 K is perceived over 35 K temperature range under light illumination condition. Furthermore, the observed photoelectrocaloric response is in correlation with the reported theoretical model. Although there are differences in quantifying the EC response obtained from the direct and indirect measurements reported in the literature, the observed light-enhanced EC effect in the BBLT system obtained from the indirect measurement establishes the light as an external stimulus to tune the EC characteristics in the ferroelectric system. In conclusion, the demonstrated photoelectrocaloric effect can be extended to other systems to exhibit needed EC responses suitable for solid-state cooling device applications.

## Supplementary Information

Supplementary Information.

## Data Availability

All data generated or analyzed during this study are included in this published article [and its supplementary information files].
