# Supplementary material for: Photoelectrocaloric effect in ferroelectric oxide
Source: Sci Rep. 2022 Apr 16;12:6390. doi: 10.1038/s41598-022-10331-8 (PMC9013360; doi:10.1038/s41598-022-10331-8)
Supplement: Supplementary file 1 — Supplementary Information. [file 41598_2022_10331_MOESM1_ESM.docx]

**Supplementary Information**

**Photoelectrocaloric effect in ferroelectric oxide**

Subhajit Pal, Manu Mohan, K. Shanmuga Priya and P. Murugavel^*^

Functional Oxides Research Group (FORG), Department of Physics, Indian Institute of Technology Madras, Chennai 600036, India





**Figure S1:** (a) *P-E* hysteresis loop measured at 10 Hz under dark and different light illumination conditions. (b) *P*_r_ value with light intensity.


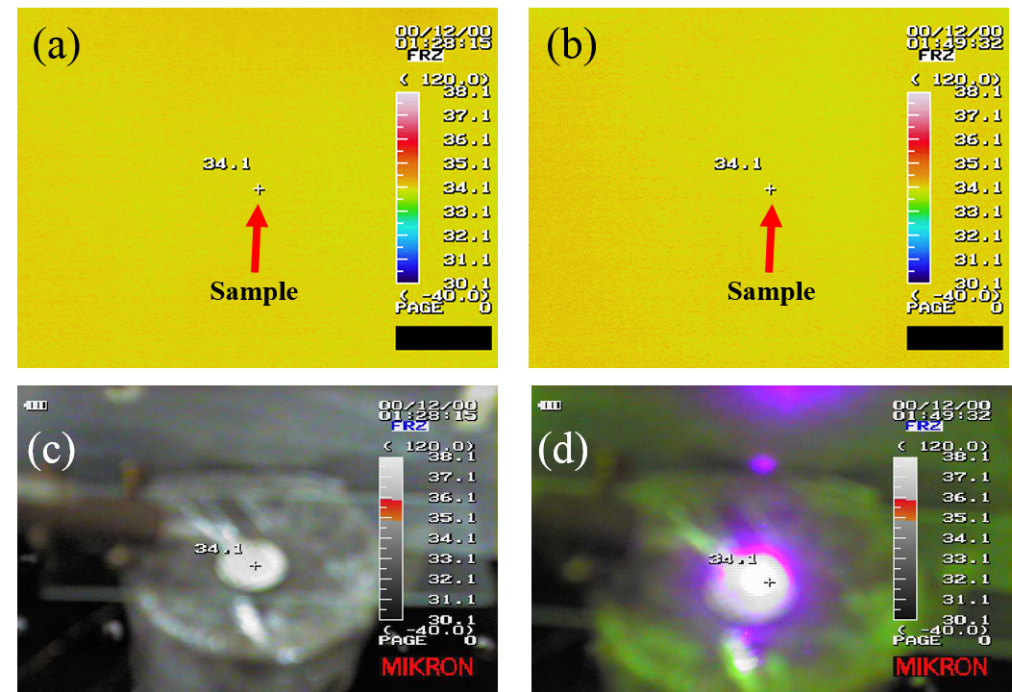


**Figure S2**: IR mode images of sample (a) normal and (b) 20 min of illumination conditions. The corresponding visible mode images are shown in (c) and (d).


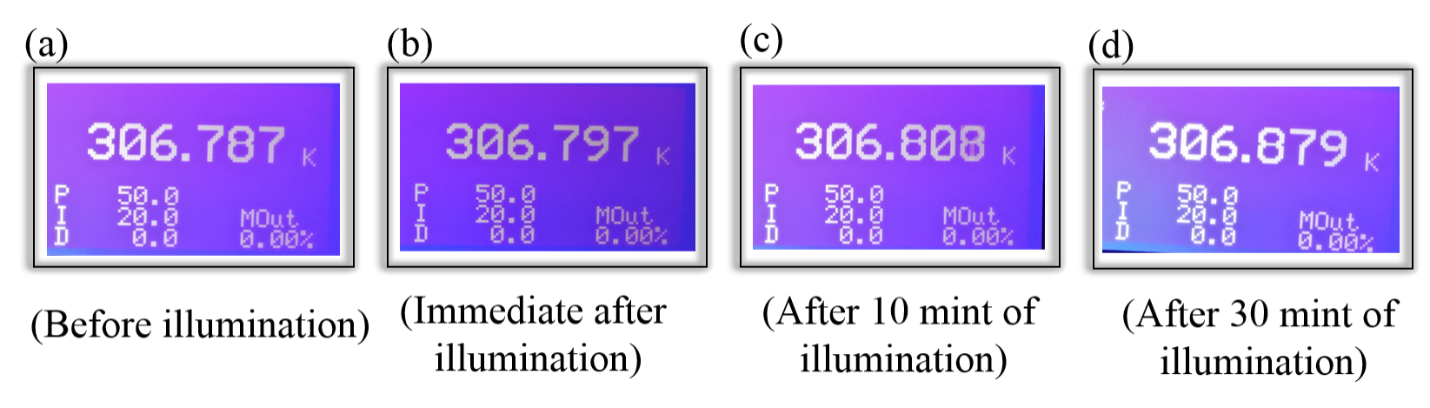


**Figure S3**: Variation in temperature under dark and different light illumination conditions.
